# Galectin-1 in Early Acute Myocardial Infarction

**DOI:** 10.1371/journal.pone.0086994

**Published:** 2014-01-31

**Authors:** Suhail Al-Salam, Satwat Hashmi

**Affiliations:** Department of Pathology, College of Medicine and Health Sciences, United Arab Emirates University, Al Ain, Abu Dhabi, United Arab Emirates; Scuola Superiore Sant’Anna, Italy

## Abstract

Myocardial infarction (MI) is the most serious manifestation of coronary artery disease and the cause of significant mortality and morbidity worldwide. Galectin-1(GAL-1), a divalent 14.5-kDa protein, is present both inside and outside cells, and has both intracellular and extracellular functions. Hypoxia inducible factor-1 alpha (HIF-1α) is a transcription factor mediating early and late responses to myocardial ischemia. Identification of the pattern of expression of GAL-1 and HIF-1α in the heart during the first 24 hours following acute MI will help in understanding early molecular changes in this event and may provide methods to overcome serious complications. Mouse model of MI was used and heart samples were processed for immunohistochemical and immunofluorescent labeling and Enzyme linked immunosorbent assay to identify GAL-1 and HIF 1α levels in the heart during the first 24 hours following MI. There was significant increase in left ventricular GAL-1 at 20 (p = 0.001) and 30 minutes (p = 0.004) following MI. There was also a significant increase in plasma GAL-1 at 4 hours (p = 0.012) and 24 hours (p = 0.001) following MI. A significant increase in left ventricular HIF-1 α was seen at 20 minutes (p = 0.047) following MI. In conclusion, we show for the first time that GAL-1 level in the left ventricle is increased in early ischemic period. We also report for the first time that HIF-1 α is significantly increased at 20 minutes following MI. In addition we report for the first time that mouse plasma GAL-1 level is significantly raised as early as 4 hours following MI.

## Introduction

Myocardial infarction (MI) is the most dreaded but likely manifestation of coronary artery disease, which is the cause of significant mortality and morbidity worldwide. Early diagnosis and timely intervention have improved outcomes and remains the cornerstone of therapy for acute MI. Understanding the very early changes that the myocardium undergoes following an ischemic event is the key to devising ways that will ultimately enable diagnosing a cardiac ischemic event before it has caused significant damage to the heart.

Galectins are a family of β-galactoside-binding lectins [1]. Fifteen members have been identified and are found to be widely distributed from lower invertebrates to mammals [2], [3]. Galectin-1 [GAL-1] is a prototypical member of the galectin family of lectins. It is a divalent 14.5-kDa protein characterized by one carbohydrate recognition domain (CRD) that can occur as a monomer or as a non-covalent homodimer consisting of subunits of one CRD [1], [4]. GAL-1 is produced by a variety of vascular, interstitial, epithelial, and immune cells [5]–[8]. GAL-1 is present both inside and outside cells, and has both intracellular and extracellular functions. The extracellular functions require the carbohydrate-binding properties while the intracellular ones are associated with protein-protein interactions [4]. GAL-1 forms lattice-like complexes with receptors that participate in recognition of cell-matrix [9]–[12]. GAL-1 lacks recognizable secretion signal sequences and does not pass along the standard endoplasmic reticulum/Golgi pathway [13]. GAL-1 is secreted through the non-classical pathway via inside-out transportation involving direct translocation across the plasma membrane and requiring unidentified integral membrane proteins and cytosolic factors [14]. In the extracellular compartment GAL-1 regulates cell-cell and cell-matrix interactions, the immune response, apoptosis, and neoplastic transformation. In the intracellular compartment it regulates cell cycle, RNA splicing and transcription [12], [15]–[19]. Intracellular GAL-1 has been shown to be present in cells nuclei and cytosols [4]. Although GAL-1 is involved in very important functions in vitro and in vivo, GAL-1 null mice are viable indicating that its presence is not critical for mammalian development or survival [11].

Studies have identified GAL-1 as hypoxia-induced protein. The hypoxic regulation of GAL-1 at mRNA and protein levels has been demonstrated in tumor biology and has the potential to be used as a prognostic marker of malignancy [20]. Under hypoxic or ischemic conditions in the brain either in vitro or in vivo, GAL-1 was found to inhibit the proliferation of astrocytes and attenuate astrogliosis. GAL-1 treatment reduced apoptosis of neurons, decreased brain infarction volume and improved neurological function induced by the ischemia, making GAL-1 a potential therapeutic target for attenuating neuronal damage and promoting recovery of brain ischemia [21]. Studies have shown that in lung tissue; the expression of GAL-1 is diffusely distributed throughout the interstitium and near to the basement membrane of vessels and airways in both normal and hypoxia-exposed mice. The difference was that the intensity of GAL-1 staining was increased in hypoxia-exposed mice, which suggest that GAL-1 may be important in adaptive responses of murine lung to chronic hypoxia [22]. The above-mentioned studies have shown that GAL-1 is regulated by hypoxia but its exact mechanism remains elusive.

Recently, Zhao et al [23] has demonstrated that hypoxia inducible factor- 1α (HIF- 1α) significantly increases GAL-1 expression in messenger RNA and protein levels in four colorectal cancer cell lines and it has been proposed that GAL-1 gene is a direct target of transcriptional factor HIF-1 α [22], [23]. HIF-1α itself is a transcription factor mediating early [24] as well as late responses to myocardial ischemia [25].

In the present study, we investigated if there is any change in the endogenous production of GAL-1 in early ischemia and its pattern of expression in the ischemic and non-ischemic cardiomyocytes. We will further elaborate on the role of HIF-1 α in the early phases of acute ischemia.

## Materials and Methods

### Animal Groups

Male C57B6/J mice were divided into 5 groups with the following time points: Group I: 20 minutes post MI (*n = 8*), Group II: 30 minutes post MI (*n = 8*), Group III: 60 minutes post MI *(n = 8),* Group IV: 4- hour post MI *(n = 8)* and Group V: 24- hour post MI *(n = 8).* Samples from sham operated animals, which are our controls, (20 minutes sham *n = 7*, 30 minutes sham *n = 7*, 60 minutes sham *n = 7*, 4- hour sham *n = 7* and 24- hour sham *n = 7*) for each mentioned time points were also studied. Samples from non-operated normal animals (Naïve *n = 7*) were also studied. Another separate group for 20 min MI *(n = 6)* and sham-operated animals *(n = 6)* were also studied.

### Ethical Approval

The Animal Research Ethics Committee of the College of Medicine and Health Sciences, UAE University have approved all experimental procedures; Protocol No.A12/10.

### Murine Model of Myocardial Infarction

All experiments were performed with the guidelines for the ‘Principles of Laboratory Animal Care’ and the ‘Guide for the care and use of laboratory animals’ published by NIH (NIH Publication No. 85-23, revised 1996), and specific national laws have been observed. C57B6/J mice (male, age: 12–16 weeks; wt: 20–25 g) were anesthetized by an intraperitoneal injection of a combination of Ketamine (100 mg/kg) and Xylazine (10 mg/kg). The mice were then intubated by transesophageal illumination using a modified 22-gauge plastic cannula and fixed on the operating pad in the supine position by taping all four extremities. The mice were connected to a mouse ventilator (Harvard apparatus Minivent Hugo Sachs Electronik) which supplied room air supplemented with 100% oxygen (tidal volume 0.2 ml/min., rate 120 strokes/min). Rectal temperature was continuously monitored and maintained within 36–37°C using a heat pad. The lead II ECG (ADInstrument multi-channel recorder interfaced with a computer running Power lab 4/30 data acquisition software) was recorded from needle electrodes inserted subcutaneously. Myocardial infarction was induced in the mice by permanently occluding the left anterior descending coronary (LAD) artery as described earlier [26]–[28].

Briefly, the chest was opened with a lateral incision at the 4^th^ intercostal space on the left side of the sternum. Next the chest wall was retracted for better visualization of the heart. With minimal manipulation, the pericardial sac was removed and the left anterior descending artery (LAD) was visualized with a stereomicroscope (Zeiss STEMI SV8). An 8-0 silk suture was passed under the LAD and ligated 1 mm distal to left atrial appendage. Occlusion was confirmed by observing immediate blanching of the left ventricle (LV) post ligation. An accompanying ECG recording showed characteristic ST-elevation, which further confirmed ischemia. The chest wall was closed by approximating the third and fourth ribs with one or two interrupted sutures. The muscles returned back to their original position and the skin closed with 4-0 prolene suture. The animal was gently disconnected from the ventilator and spontaneous breathing was seen immediately. Postoperative analgesic (Butorphanol 2 mg/kg, s/c, 6 hourly) was given at the end of the procedure. Sham operated mice underwent exactly the same procedure described above, except that the suture passed under the LAD is left open and untied. Sham operated mice groups are our control and they exactly follow the same time points of corresponding MI groups. According to the experimental protocol, mice were sacrificed after induction of myocardial infarction with desired time of ischemia. The method of euthanasia started with intraperitoneal injection of anesthetic drugs, which included a combination of Ketamine (100 mg/kg) and Xylazine (10 mg/kg). When the mouse was completely anesthetized, skin and chest wall were re-opened. Blood was collected in EDTA vacutainers and the heart was resected, washed in ice cold phosphate buffered saline (PBS), the right ventricle and both atria were dissected away and LV was immediately frozen in liquid nitrogen and later stored in −80°C freezer. Collected blood was centrifuged at 3000 RPM for 15 minutes. The plasma was collected, alliquoted and stored at −80°C until further analysis. Heart samples from the same time point following LAD ligation were also fixed in 10% buffered formal-saline for 24 hours.

### Sample Processing for Protein Extraction

Total protein was extracted from heart samples by homogenizing with lysis buffer and collecting the supernatant after centrifugation. For total cell lysate, the left ventricular heart along with the septum samples were thawed, weighed and put in cold lysis buffer containing 50 mM Tris, 300 mM NaCl, 1 mM MgCl_2,_ 3 mM EDTA, 20 mM β-glycerophosphate, 25 mM NaF, 1% Triton X-100, 10%w/v Glycerol and protease inhibitor tablet (Roche Complete protease inhibitor cocktail tablets). The hearts were homogenized on ice by a homogenizer (IKA T25 Ultra Turrax). The samples were then centrifuged at 14000 RPM for 15 minutes at 4 ^o^ C, supernatant collected, alliquoted and stored at −80°C until further analysis. Nuclear and cytoplasmic protein extraction was done following the protocol described elsewhere [29]. Briefly, the heart LV were thawed on ice, weighed and homogenized on ice with buffer containing Tris HCl 10 mmol/l, CaCl_2_ 3 mmol/l, MgCl_2_ 2 mmol/l, EDTA 0.1 mmol/l, Phenylmethanesulfonyl fluoride (PMSF) 0.5 mmol/l, Sucrose 0.32 mmol/l, Dithiothreitol (DTT) 1 mmol/l, Nonidet P-40 (NP-40) 0.5% and Protease inhibitor cocktail 1%. The homogenates were centrifuged at 800 g for 10 minutes. The supernatant removed and kept as cytoplasmic fraction. The pellet was washed twice with homogenization buffer without NP-40 and resuspended with a low salt buffer containing HEPES 20 mmol/l, MgCl_2_ 1.5 mmol/l, KCl 20 mmol/l, EDTA 0.2 mmol/l. Glycerol 25%, PMSF 0.5 mmol/l and DTT 0.5 mmol/l. After incubation on ice for 5 minutes, an equal volume of high salt buffer containing HEPES 20 mmol/l, MgCl_2_ 1.5 mmol/l, KCl 800 mmol/l, EDTA 0.2 mmol/l. Glycerol 25%, PMSF 0.5 mmol/l, DTT 0.5 mmol/l, NP-40 1% and protease inhibitor cocktail 1%, was added, the mixture incubated on ice for 30 minutes, centrifuged at 14000 g for 15 minutes at 4°C and supernatants saved as the nuclear fraction. Total protein concentration was determined by BCA protein assay method (Thermo Scientific Pierce BCA Protein Assay Kit).

### Sample Processing for Histology

Hearts were excised, washed with ice-cold PBS and weighed. Each heart was sectioned, cassetted and fixed directly in 10% buffered formalin. Sections were dehydrated in increasing concentrations of ethanol, cleared with xylene and embedded with paraffin. Three-µm sections were prepared from paraffin blocks and stained with haematoxylin and eosin. The stained sections were evaluated by the histopathologist that participated in this project.

#### Immunohistochemistry

Five- µm sections were cut, de-waxed with xylene and rehydrated with graded alcohol. The slides were then placed in a 0.01 M citrate buffer solution (pH = 6.0) and pre-treatment procedures to unmask the antigens was performed in a microwave oven for 10 minutes. Sections were treated with peroxidase and protein block for 60 min each and then incubated overnight with anti- GAL-1 (rabbit anti-mouse polyclonal antibody 1∶2500, Davids Biotechnologie GmbH, Germany), anti- HIF 1 α (rabbit anti-mouse polyclonal antibody 1∶300, Davids Biotechnologie GmbH, Germany), anti-cleaved caspase 3(Rabbit polyclonal, ASP 175, Cell Signaling Technology, USA), anti-Bcl2 (Mouse monoclonal,SP66,Cell Marque, USA )and anti-ki67 (Rabbit monoclonal, SP6, Cell Marque, USA) antibodies at 4°C. After conjugation with primary antibodies, sections were incubated with biotin-labeled secondary antibody (Thermo Scientific, USA) for 20 minutes at room temperature. Finally, sections were incubated with streptavidin–peroxidase complex for 20 minutes at room temperature (Thermo Scientific, USA), DAB chromogen (Thermo Scientific, USA) added and counter staining done with haematoxylin. Appropriate positive controls were used. For negative control, the primary antibody was not added to sections and the whole procedure carried out in the same manner as mentioned above.

#### Immunofluorescent labeling

5 µm sections were deparaffinized with xylene and rehydrated with graded alcohol. Sections were placed in EnVision™ FLEX Target Retrieval Solution with a high PH (PH 9) (DAKO Cytomation, Denmark) in a water bath at 80°C for one hour. Later they were incubated with anti-HIF-1α (rabbit anti-mouse polyclonal antibody, Davids Biotechnologie GmbH, Germany), anti-GAL-1 (rabbit anti-mouse polyclonal antibody, Davids Biotechnologie GmbH, Germany), and anti-caspase-3(Rabbit polyclonal, (CPP32) Ab-4, Thermo Scientific™ Lab Vision, USA) overnight at room temperature. Sections were subsequently incubated with Donkey anti-rabbit Ig conjugated-Rhodamine (Santa Cruz Biotechnology, USA, 1∶50), or with donkey anti-rabbit Alexa Fluor 488, (Invitrogen, USA, 1∶50). Finally, sections were mounted in water-soluble mounting media and viewed with Olympus Fluorescent microscope. Immunofluorescent double labeling for GAL-1 with HIF-1α, desmin, CD31, CD68, and HIF-1α, with CD31 and CD 68 were done on heart sections in the same way as described above. The first primary antibody is labeled with fluorescein; the second primary antibody is added afterwards followed by fluorescein label with different excitation color. Appropriate positive control sections were used. For negative control, the primary antibody was not added to sections and the whole procedure carried out in the same manner as mentioned above.

#### Morphometric analysis

Morphometric analysis of GAL-1 and HIF-1 α expression in cardiomyocytes, endothelial cells and neutrophil polymorphs was done at different time points following ligation of LAD using ImageJ software (http://rsbweb.nih.gov/ij/). GAL-1 labeling was determined by counting the number of cardiomyocytes, endothelial cells and neutrophil polymorphs in 10 randomly-selected fields in the left ventricle. For GAL-1 labeling, cells were considered positive when there was a cytoplasmic and/or nuclear staining pattern. The labeling index for cardiomyocytes, endothelial cells and neutrophils were expressed as the percentage of labeled cells against the total number of cell enumerated. For HIF-1 α labeling, cells were considered positive when there was a nuclear staining pattern. The labeling index for cardiomyocytes, endothelial cells and neutrophils were expressed in the same way as done for GAL-1. Neutrophil polymorphs were counted only in 24h post MI time point.

### Enzyme Linked Immunosorbent Assay

Left ventricular myocardial concentration of GAL-1 and HIF-1 α and plasma levels of GAL-1 was determined using DuoSet enzyme linked immunosorbent assay (ELISA) Development kit (R&D Systems, Minneapolis, MN, USA) for sandwich ELISA, using standard procedure according to the manufacturer’s instructions. The levels were normalized to total protein concentrations.

Briefly, 96-well plates (Nunc-Immuno Plate MaxiSorp Surface (NUNC Brand Products, A/S, Roskilde, Denmark), were coated with antibody specific for GAL-1 and HIF-1 α. Biotinylated detection antibody and streptavidin conjugated horseradish peroxidase were used for detection of captured GAL-1 and HIF-1 α. Captured GAL-1 and HIF-1 α were visualized using tetramethylbenzidine (TMB)/hydrogen peroxide. Absorbance readings were made at 450 nm, using a 96-well plate spectrophotometer (BioTek ELx800). GAL-1 and HIF-1 α levels in samples were determined by interpolation from a standard curve.

### Bioinformatic Analysis

We used the bioinformatic analysis program Clustalw2 available on the Internet (http://www.ebi.ac.uk/Tools/msa/clustalw2/) to find out the core HIF-binding motif ‘RCGTG’, the Hypoxia response element (HRE) [30] in the promoter region of the mouse and human GAL-1 gene.

### Statistical Analysis

Statistical analysis is done using IBM SPSS Statistics version 20. Data are presented in mean ± S.E. Statistically significant differences (p<0.05) are calculated between acute MI group and corresponding sham-operated group for each time point by Student t test. One-way ANOVA is used to test for differences among the various time points within the MI groups. Chi square test is used to assess differences in the expression of GAL-1 and HIF-1 α in cardiomyocytes & endothelial cells at different time points following MI for morphometric analysis.

## Results

### Electrocardiographic Study

**Figure 1:**
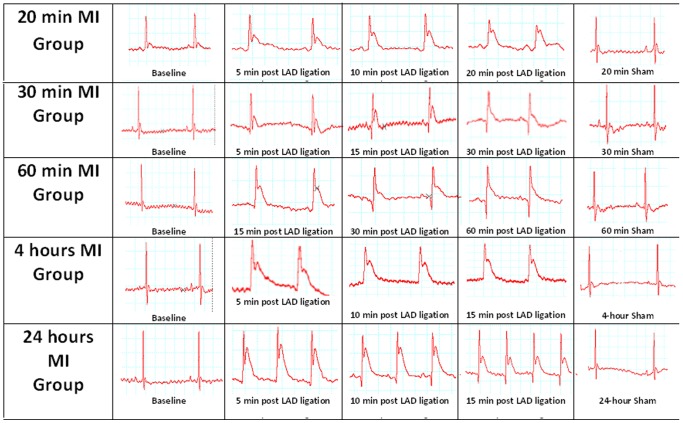
Electrocardiography of the heart at 20 minutes, 30 minutes, 60 minutes, 4-hour and 24-hour following MI. Representative ECG of 20 minutes, 30 minutes, 60 minutes, 4 hours and 24 hours post MI groups showing ST elevation compared to normal ECG in sham operated groups.

The ECG records for mice groups; 20 minutes, 30 minutes, 60 minutes, 4 hours and 24 hours following permanent ligation of LAD show persistent ST elevations. The ECG for 20 min, 30 min and 60 min post LAD ligation groups were monitored for the whole duration of time whereas for 4 hours and 24 hours post MI groups it was monitored for the first 15 minutes (**Fig. 1**). We have used persistent elevation of ST- segment in the ECG as a control for selection of animals to be included in our study.

### HIF-1 α in Heart Tissue

**Figure 2:**
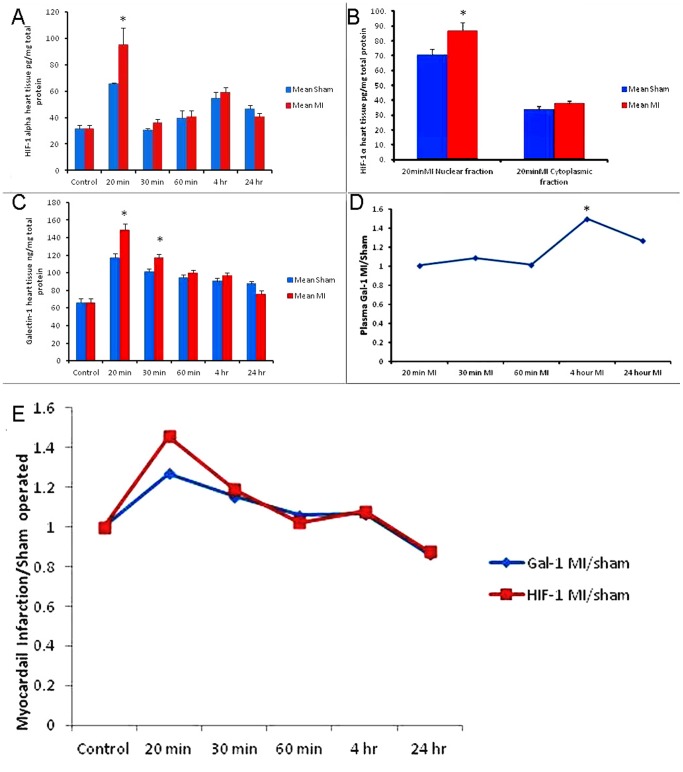
Left ventricular galectin-1 and HIF-1 α concentration and plasma concentration of galectin-1 following MI. **A.** The graph represents HIF-1 α concentrations at 20 min, 30 min, 60 min, 4 hours and 24 hours post myocardial infarction with corresponding sham operated groups in C57BL6 mouse left ventricle. Control represents non-operated normal animal heart. *Shows p<0.05. **B.** HIF-1 α levels in nuclear and cytoplasmic extracts of LV mouse heart at 20 minutes following MI. *Shows p<0.05. **C.** The graph represents left ventricular galectin-1 concentrations at 20 min, 30 min, 60 min, 4 hours and 24 hours post myocardial infarction with corresponding sham operated groups in C57BL6 mouse heart. Control represents non-operated normal animal heart. *Shows p<0.05. **D.** The graph represents ratio of plasma galectin-1 concentration in myocardial infarction to plasma galectin-1 concentration in sham operated mice C57BL6 mice. 4 hours and 24 hours post myocardial infarction show significant difference from sham operated mice. *Shows p<0.05. **E.** Pattern of Galectin-1 and HIF-1 α in the heart from 20 min post MI till 24 hour post MI time points, showing similar pattern of expression during the first 24-hour following MI.

HIF-1 α concentration in LV tissue of naïve group is 31.97±2.18 pg/mg. HIF-1 α protein concentration shows a significant increase in the LV at 20 minutes following MI group compared to sham operated control group (95.5±12.4 vs 65.6±0.7 pg/mg, ***P*** = 0.047*) (**Table 1**) (**Fig. 2 A**). HIF-1 α values in the MI groups are higher than corresponding sham operated groups at 30 minutes, 60 minutes and 4-hour groups but show no statistical significance (36.34±2.62 vs 30.54±1.42 pg/mg, 40.85±4.6 vs 40.01±5.15 pg/mg, and 59.35±3.22 vs 54.98±4.26 pg/mg,). The 24-hour HIF-1 α level in sham-operated group appears to be higher than the MI group but does not reach statistical significance (46.65±2.42 vs 40.75±2.61 pg/mg). HIF-1 α value in LV in all MI groups is higher than the baseline naïve control group.

**Table 1:** HIF-1 α levels in pg/mg of total protein at different time points post myocardial infarction.

Groups	Number	Mean(pg/mg)	Std.Dev.	Std.Error	p value
Naive	7	31.97	5.78	2.18	
20 min MI	8	95.49	35.09	12.41	0.047*
20 min sham	7	65.58	1.86	0.70	
30 min MI	8	36.34	7.39	2.62	0.103
30 min sham	6	30.54	3.47	1.42	
60 min MI	8	40.85	12.98	4.59	0.904
60 min sham	7	40.01	13.61	5.15	
4- hour MI	8	59.35	9.12	3.22	0.421
4- hour sham	7	54.98	11.28	4.26	
24 hour MI	7	40.75	6.89	2.61	0.123
24-hour sham	7	46.65	6.41	2.42	

* shows p<0.05.

The pattern of HIF-1 α protein expression in the LV after permanent ligation of LAD shows a transient significant peak at 20 minutes following MI, which goes down afterwards but still remains high compared to the naïve control animal group (**Table 1**) (**Fig. 2A**).

As we observed a peak of HIF-1 α at 20 minutes post MI time point, so we did a separate experiment for 20 minutes time point to measure the HIF-1 α concentration in the nuclear and cytoplasmic extracts of the LV. The results show a significant rise of HIF-1 α concentration in the nuclear extract of the MI group compared to the sham operated group (87.84±5.41 vs 70.75±3.72 pg/mg, p = 0.03*) **(** **Table 2** **) (** **Fig. 2 B**).

**Table 2:** HIF-1 α levels in pg/mg total protein in nuclear and cytoplasmic fractions of left ventricular heart tissue at 20 min post myocardial infarction.

HIF-1α (LV)	Groups	Number	Mean	Std. Deviation	Std. Error	P value
Nuclear fraction pg/mg	20 min MI	6	87.05	13.24	5.41	0.032*
	20 min Sham	6	70.75	9.11	3.72	
Cytoplasmic fraction pg/mg	20 min MI	6	38.16	3.60	1.47	0.125
	20 min Sham	6	34.11	4.70	1.92	

* shows p<0.05.

There was a statistically significant difference between the different time points within the MI groups as determined by one-way ANOVA (*F* (4,34) = 14.672, *p* = .000). Tukey post-hoc test showed that the 20 min post MI HIF-1 α level (M = 95.48, 95% CI [66.15, 24.82]) is significantly higher than 30 minutes (M = 36.34, 95% CI [30.15, 42.53], *p* = 0.00), 60 minutes (M = 40.85, 95% CI [30.00, 51.7], *p* = 0.00), 4 hours(M = 59.35, 95% CI [51.72, 66.98], *p* = 0.003), and 24 hours post MI group (M = 40.75, 95% CI [34.37, 47.13], *p* = 0.00).

**Figure 3:**
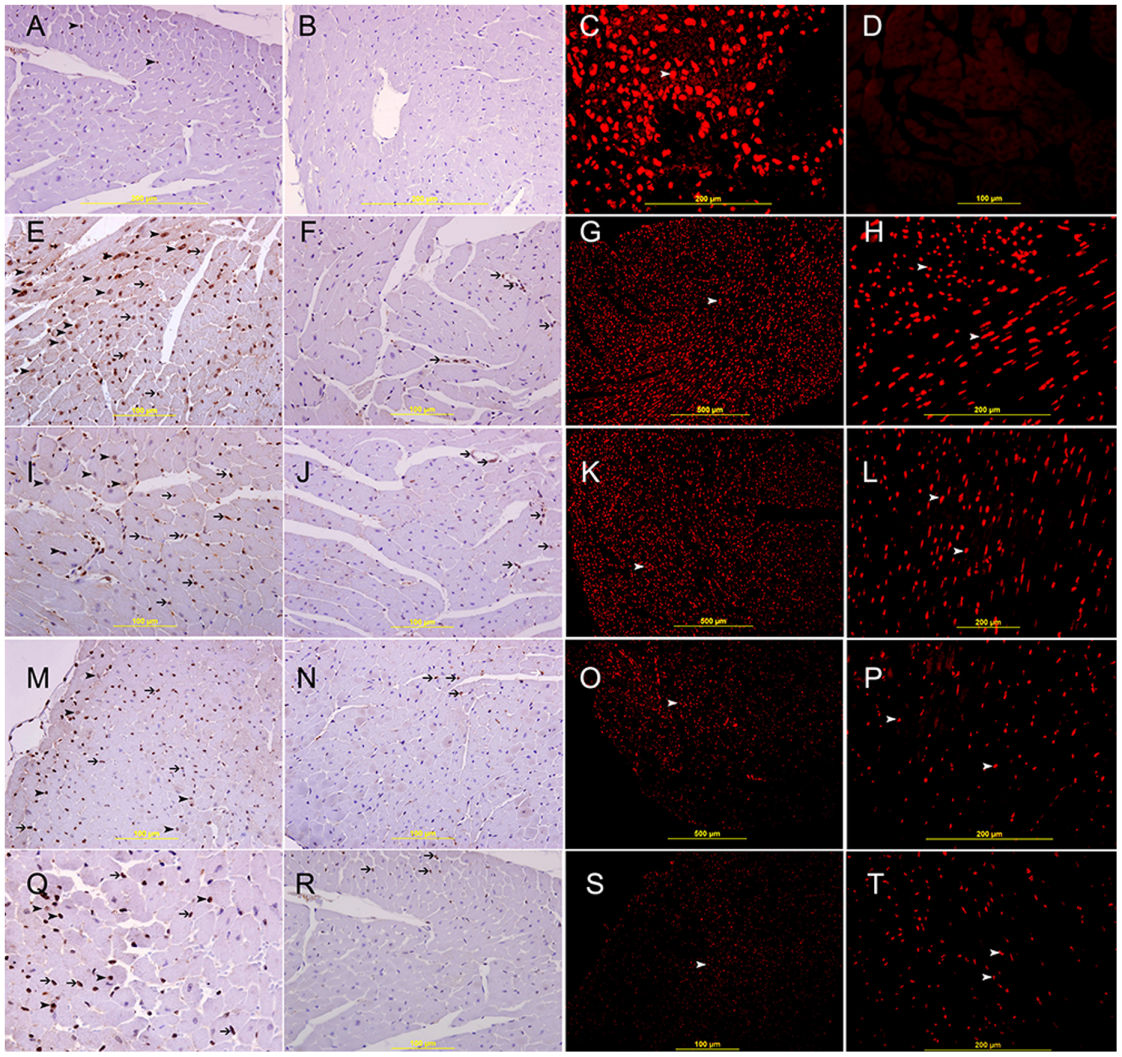
HIF-1 α expression of the heart. **A.** Representative section of naïve heart showing nuclear expression of HIF-1 α by few endothelial cells (arrow head), streptavidin- biotin immunoperoxidase method. **B.** Negative control section showing no HIF-1 α staining, streptavidin- biotin immunoperoxidase method. **C.** Positive control section of mouse placenta showing nuclear staining of HIF-1 α by trophoblastic cells, Rhodamine, immunofluorescent technique. **D.** Negative control section for HIF-1 α. Rhodamine, immunofluorescent technique. E,I,M&Q shows representative sections from the anterior wall of left ventricle in the area supplied by LAD 20 min, 30 min, 60 min and 4 hours following ligation of LAD, showing variable nuclear staining of HIF-1 α by cardiac myocytes at different time points (arrow head) and endothelial cells (thin arrow), streptavidin- biotin immunoperoxidase method. F,J,N&R shows their corresponding Sham operated hearts showing low nuclear expression of HIF-1 α by few endothelial cells (thin arrow), streptavidin- biotin immunoperoxidase method. Low and high power views of the left ventricle 20 min (G&H), 30 min (K&L), 60 min (O&P) and 4 hours (S&T) following ligation of LAD showing high nuclear staining of HIF-1 α by cardiac myocytes (arrow head), Rhodamine, immunofluorescent technique.

**Figure 4:**
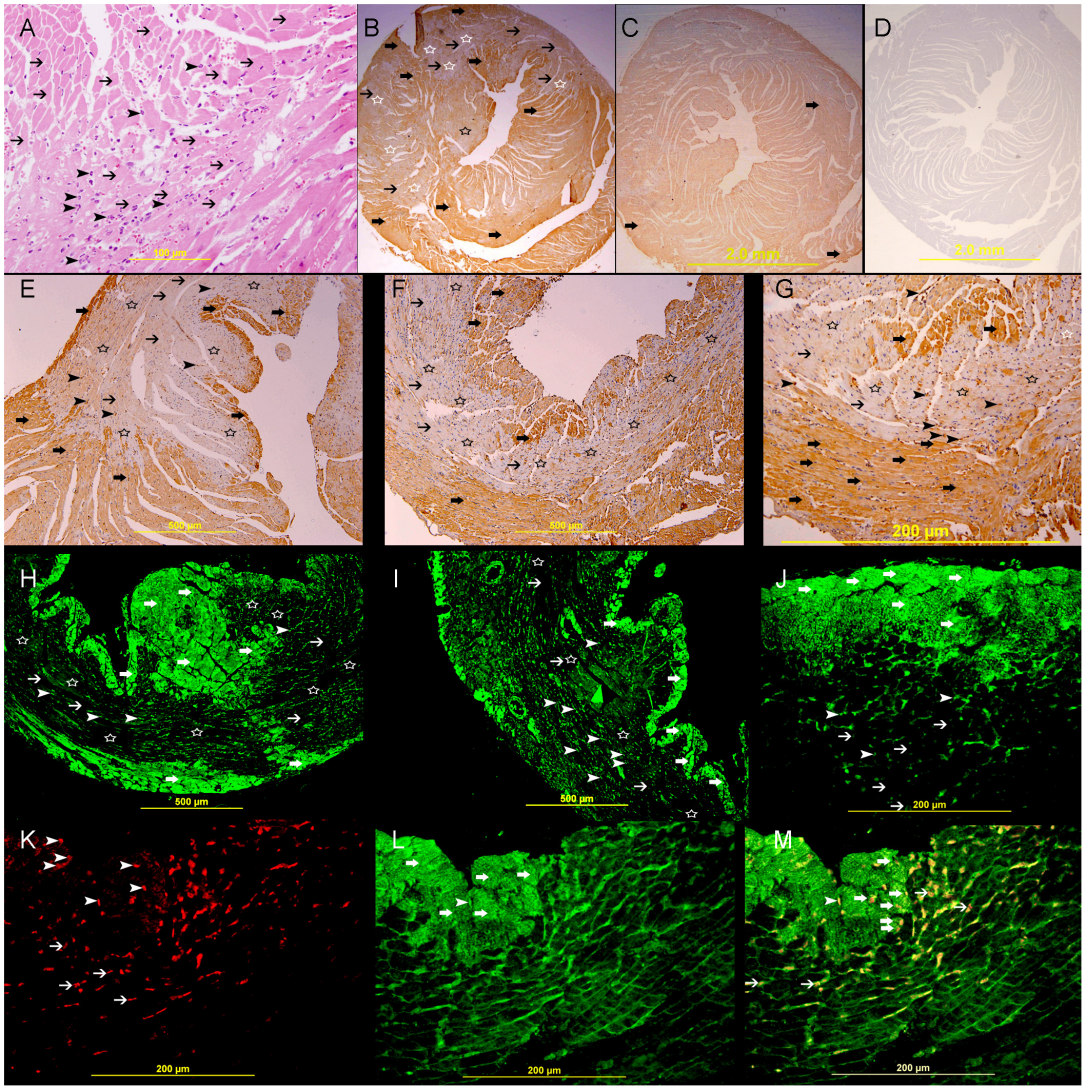
Galectin-1 and HIF-1 α expression 24 hours following ligation of LAD. A. A high power view of left ventricle in an area supplied by LAD showing coagulative necrosis (thin arrows) accompanied by heavy neutrophil polymorphs infiltration (arrow heads), H&E. B. A low power view of the heart showing areas of high expression of galectin-1 (thick arrows) surrounding areas of no expression (star shape) in the left ventricle and interventricular septum in the area supplied by LAD, streptavidin- biotin immunoperoxidase method. C. Sham operated heart showing lower expression of galectin-1 in the left ventricle and right ventricle (thick arrows), streptavidin- biotin immunoperoxidase method. D. Negative control section of the heart showing absence of staining of galectin-1. E, F&G. Representative section of the left ventricle from area supplied by LAD showing high cytoplasmic expression of galectin-1 by cardiac myocytes (thick arrow), surrounding areas of no expression (thin arrow). Many neutrophil polymorphs are also showing cytoplasmic expression of Galectin-1(arrow head), Streptavidin- biotin immunoperoxidase method. H, I&J. Representative section from left ventricle showing a well demarcated area of high cytoplasmic expression of galectin-1 by cardiac myocytes (thick arrow) surrounding areas of no expression (thin arrow), many neutrophil polymorphs (arrow head) show cytoplasmic expression of Galectin-1, Alexa Fluor 488 immunofluorescent technique. K. Representative section from left ventricle showing nuclear expression of HIF-1 α by cardiac myocytes (arrow head) and neutrophil polymorphs (thin arrow), Rhodamine, immunofluorescent technique. L. Representative section from left ventricle showing cytoplasmic expression of Galectin-1 by cardiac myocytes (thick arrow), endothelial cells (arrow head) and neutrophil polymorphs (thin arrows), Alexa Fluor 488 immunofluorescent technique. M. Co-localization of Galectin-1 and HIF-1 α in cardiac myocytes (thick arrow), endothelial cell (arrow head) and neutrophil polymorphs (thin arrow), Alexa Fluor-Rhodamine immunofluorescent technique.

The expression of HIF-1α is predominantly seen in the nuclei of endothelial cells (**Fig. 3** **A**) in the naïve heart. The expression of HIF-1 α in the nuclei of cardiomyocytes is very low and in few cells in naïve heart. There is a higher nuclear expression of HIF-1 α by cardiac myocytes and endothelial cells in the area supplied by LAD artery at 20 **(** **Fig. 3 E, G, H**) and 30 minutes (**Fig. 3 I, K, L**), groups when compared with 60 minute (**Fig. 3 M, O, P** ****), 4 hours (**Fig. 3 Q, S, T**), and 24 hours (**Fig. 4K** **)** following MI groups and all sham operated groups. In 60 minute (**Fig. 3 M, O, P**), 4 hours (**Fig. 3 Q, S, T**), we noticed a decrease in the number of cardiac myocytes that expresses HIF-1 α and most of the cells that express HIF-1 α are endothelial cells. In 24 hours post MI group the expression of HIF-1α is in the nuclei of few cardiomyocytes and more endothelial cells in areas surrounding the infarction while cardiomyocytes in the center of infarction does not show any expression of HIF-1α (**Fig. 4K**). On the contrary, many infiltrating neutrophil polymorphs in the center of infarction show nuclear expression of HIF-1 α (**Fig. 4K**). As early as 60 minutes following MI we can identify an area of infarcted cardiomyocytes that they do not express HIF-1 α (**Fig. 3 M**) this area is more obvious at 4 hours **(** **Fig. 3 Q** **)** and 24 hours following MI (**Fig. 4K**).

### Galectin-1 in the Heart Tissue

GAL-1 concentration in LV tissue of naïve group is 66.32±4.69 ng/mg. GAL-1 concentration in LV tissue is significantly increased at 20 minutes (148.91±6.65 vs. 117.20±4.45 ng/mg ***P*** = 0.001*) and 30 minutes (117.45±3.49 vs 101.79±2.63 ng/mg, ***P*** = 0.004*) post MI groups compared to corresponding sham operated control groups **(** **Table 3** **)** (**Fig. 2 C**). GAL-1 values at 60 minutes and 4 hours post MI groups are higher than their corresponding sham operated control groups but do not reach statistical significance (100.43±3.05 vs 94.99±2.76 ng/mg, *P* = 0.208 and 97.01±3.31 vs 91.07±3.26 ng/mg, *P* = 0.224). At 24-hour post MI group, GAL-1 concentration in sham-operated control group is a bit higher than its corresponding MI group (85.24±2.42 ng/mg vs 75.71±3.87) but shows no statistical significance. GAL-1 values in LV in all MI groups are higher than the baseline naïve control group.

**Table 3:** Galectin-1 levels in ng/mg of total protein at different time points post myocardial infarction.

Groups	Number	Mean(ng/mg)	StdDev	StdError	p value
Naive	7	66.32	12.39	4.69	
20 min MI	8	148.91	18.81	6.65	0.001*
20 min sham	8	117.20	12.57	4.45	
30 min MI	8	117.45	9.88	3.49	0.004*
30 min sham	7	101.79	6.97	2.63	
60 min MI	8	100.43	8.63	3.05	0.208
60 min sham	7	94.99	7.29	2.75	
4 -hour MI	8	97.01	9.36	3.31	0.224
4- hour sham	7	91.07	8.63	3.26	
24- hour MI	8	75.71	10.94	3.87	0.079
24 -hour sham	6	85.24	5.92	2.42	

* shows p<0.05.

There was a statistically significant difference between the different time points within the MI groups as determined by one-way ANOVA (F (4,35) = 40.693, *p* = .000). A Tukey post-hoc test revealed that the 20 minutes post MI GAL-1 level (M = 148.9, 95% CI [133.18, 164.6]) is significantly higher than the 30 minutes (M = 117.4, 95% CI [109.2, 125.72], *p* = 0.00), 60 minutes(M = 100.43.9, 95% CI [93.22, 107.65], *p* = 0.00), 4 hour (M = 97.01, 95% CI [89.18, 104.84], *p* = 0.00) and 24 hour post MI group (M = 75.71, 95% CI [66.57, 84.86], *p* = 0.00 ). 30 minutes post MI GAL-1 value is also higher than the 4 hour (*p* = 0.015), and 24 hours time point (*p* = 0.00). 30 minutes post MI GAL-1 value is also higher than 60 minutes post MI group but not reach statistical significance (*p* = 0.058). 60 minutes post MI group value is significantly higher than the 24 hour MI group (*p* = 0.002) and 4 hour MI level is significantly higher than 24 hour post MI time point (*p* = 0.01).

**Figure 5:**
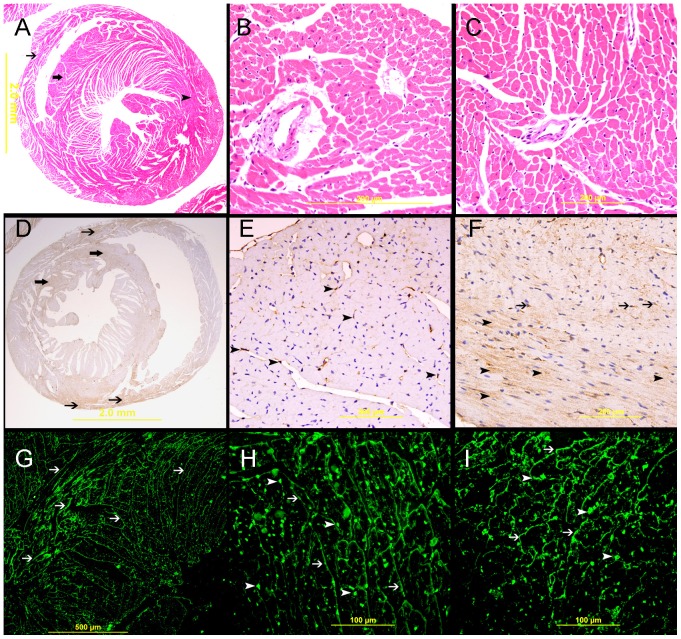
Galectin-1 expression in naïve heart. I, Naïve heart, A. Low power view of the heart showing the left ventricle (arrow head), interventricular septum (thick arrow) and right ventricle wall (thin arrow), H&E stain. B&C are representative sections of the left and right ventricles respectively, H&E stain. D. showing low and focal expression of galectin-1 in the wall of the right ventricle (thin arrow) and the interventricular septum (thick arrow). E. A representative section of left ventricle showing mild expression of galectin-1 by endothelial cells (arrow head). F. A representative section of right ventricle showing low cytoplasmic expression of galectin-1 by cardiac myocyte (arrow head) and endothelial cells (thin arrow). G, H&I, immunofluorescent labeling of sections of the heart showing a net-like pattern of galectin-1 staining of cardiac myocytes (thin arrow) and cytoplasmic staining of endothelial cells (arrow head).

**Figure 6:**
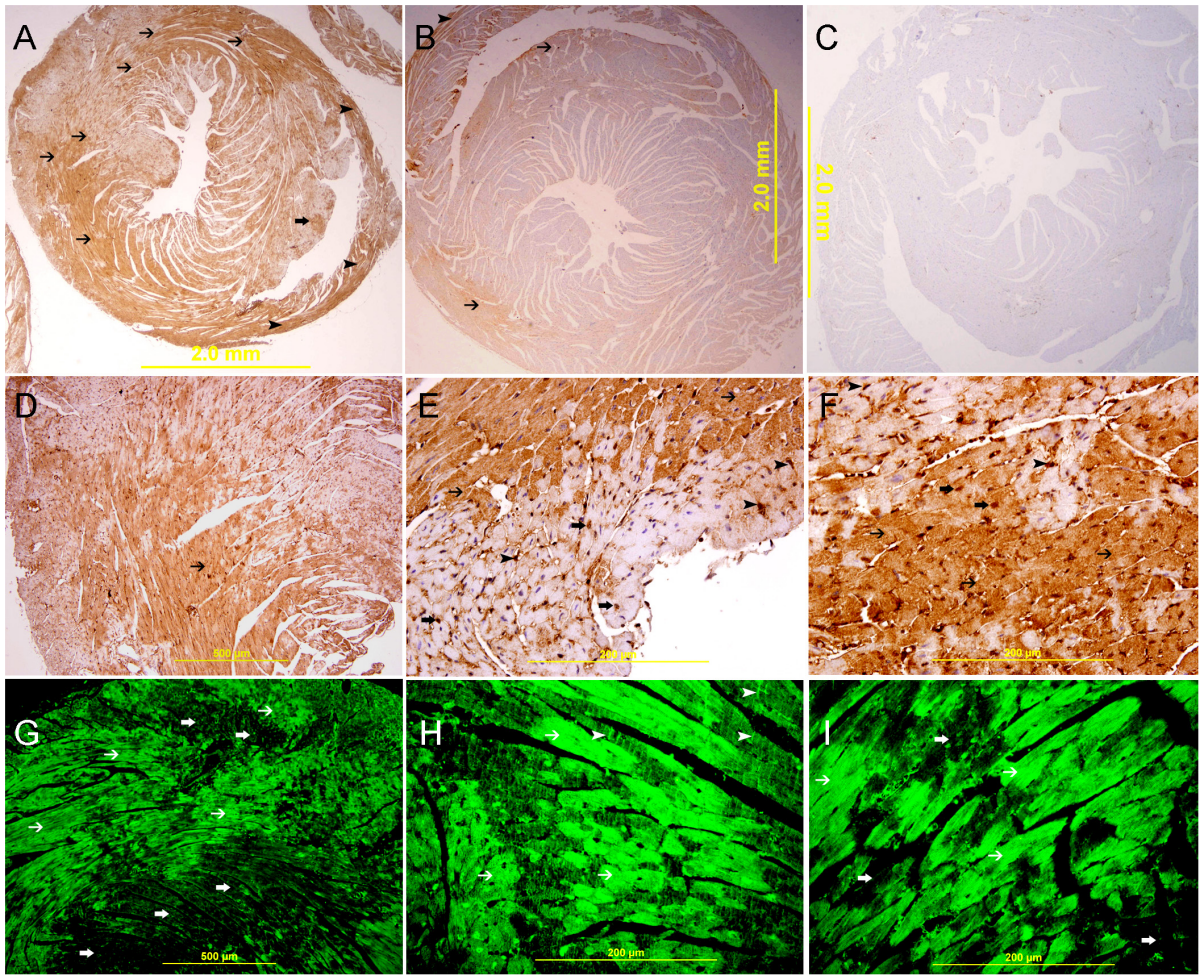
Galectin-1 expression 20 minutes following ligation of LAD. A. A low power view of the heart showing a high expression of galectin-1 in the anterior wall of left ventricle in the area supplied by LAD (thin arrow) and the interventricular septum (thick arrow). There is also increase in the expression of galectin-1 in the right ventricle (arrow head), streptavidin- biotin immunoperoxidase method. B. Sham operated heart showing lower expression of galectin-1 in the left ventricle and right ventricle (thin arrow), streptavidin- biotin immunoperoxidase method. C. Negative control section of the heart showing absence of galectin-1 staining. D, E&F. Representative sections of the left ventricle from area supplied by LAD showing high cytoplasmic expression of galectin-1 by cardiac myocytes (thin arrow D). High power views show a well demarcated area of high cytoplasmic expression of galectin-1 by cardiac myocytes (thin arrow) and endothelial cells (arrow head, E&F), There is also nuclear expression of galectin 1 by cardiac myocytes (thick arrow E&F),streptavidin- biotin immunoperoxidase method. G, H&I. Representative section from left ventricle shows a well demarcated area of high cytoplasmic expression of galectin-1 by cardiac myocytes (thin arrow).There is also high expression of galectin-1 in the Z bands (arrow head), Alexa Fluor 488 immunofluorescent technique.

**Figure 7:**
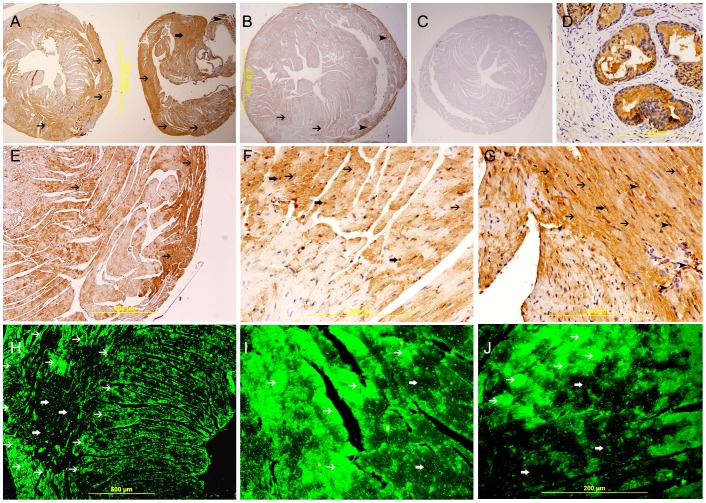
Galectin-1 expression 30 minutes following ligation of LAD. A. A low power view of the heart showing a high expression of galectin-1 in the anterior wall of left ventricle in the area supplied by LAD (thin arrow) and the interventricular septum (thick arrow). There is also increase in the expression of galectin-1 in the right ventricle (arrow head), streptavidin- biotin immunoperoxidase method. B. Sham operated heart showing lower expression of galectin-1 in the left ventricle and right ventricle (thin arrow), streptavidin- biotin immunoperoxidase method. C. Negative control section of the heart showing absence of staining of galectin-1. D. A galectin-1 positive control section from prostate gland showing cytoplasmic expression of galectin- 1 by prostatic acini. E, F & G. Representative sections of the left ventricle from areas supplied by LAD showing high cytoplasmic expression of galectin-1 by cardiac myocytes (thin arrow), streptavidin- biotin immunoperoxidase method. H, I&J. Representative sections from left ventricle showing a well demarcated area of high cytoplasmic expression of galectin-1 by cardiac myocytes (thin arrow), Alexa Fluor 488 immunofluorescent technique.

In normal heart, GAL-1 is mainly seen to be expressed by cardiomyocytes and endothelial cells (**Fig. 5**). The expression is more pronounced in the right ventricle as compared to the left ventricle (**Figure 5** D, E, F). In the left ventricle, GAL-1 is mainly expressed by endothelial cells and few cardiomyocytes **(** **Fig. 5E** **)**. A very characteristic staining pattern is seen in immunofluorescent labeling of GAL-1, which gives a mesh-like staining pattern of myocardium **(** **Fig. 5 G, H, I** **)**. There is an increase in the expression of GAL-1 in the LV as compared with sham operated heart sections. In MI groups, the expression of GAL-1 is well demarcated in the area supplied by LAD artery at 24- hour, 20, 30, 60 minutes and 4- hour following MI (**Fig. 4** **,** **6** **,** **7** **,** **8** **&** **9** **respectively**). Both cardiac myocytes and endothelial cells show high expression of GAL-1 (**Fig. 6E** **,** **7F** **,** **8F** **,** **9F**). In addition, there are foci of low or no expression of GAL-1 surrounded by areas of high expression (**Fig. 4** **,** **6** **,** **7** **,** **8** **&** **9**).The number of cardiac myocytes that express GAL-1 is decreased as the time following ligation is increased. This phenomenon is also seen but to a lesser extent in endothelial cells. Hence, areas of low or no expression of GAL-1 increase in size and become more demarcated as we reach 4 hours and then 24 hours following MI (**Fig. 4 E, F, G** **& 9 D, E, F, G, H**). In 24 hours post MI, the GAL-1 expression is seen in areas surrounding the infarction (**Fig. 4 E, F, G, H& I**). There is no expression of GAL-1 in the dead cardiomyocytes hence, these areas are very sharply demarcated (**Fig. 4 E, F, G, H& I**). Neutrophil polymorphs, which are abundant at 24 hours post MI group, also show high expression of GAL-1 (**Fig. 4 G& H**). GAL-1 show many patterns of staining in the heart at different time points following MI which include diffuse cytoplasmic staining (**Fig. 7 G& I**), Z bands staining (**Fig. 6H**), cell membrane staining (**Fig. 5 G, H, I** **and** **Fig. 10Q**) and nuclear staining (**Fig. 6 E&F**).

**Figure 8:**
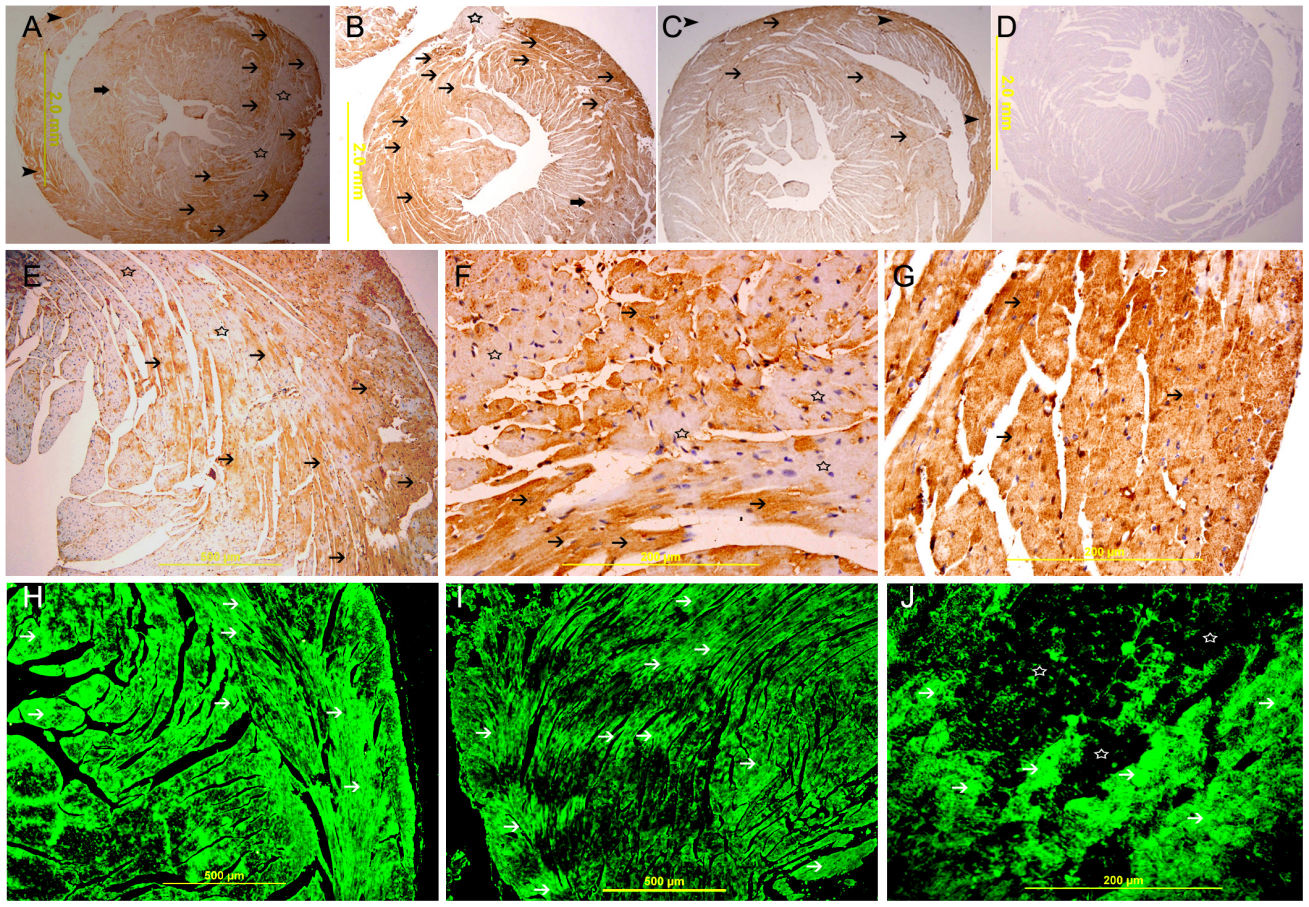
Galectin-1 expression 60 minutes following ligation of LAD. A& B, A low power view of the heart showing a high expression of galectin-1 in the anterior wall of left ventricle in the area supplied by LAD (thin arrow) and the interventricular septum (thick arrow). There is also increase in the expression of galectin-1 in the right ventricle (arrow head), streptavidin- biotin immunoperoxidase method. C. Sham operated heart showing low expression of galectin-1 in the left ventricle and right ventricle (thin arrow), streptavidin- biotin immunoperoxidase method. D. Negative control section of the heart showing absence of staining of galectin-1. E, F & G, Representative sections of the left ventricle from areas supplied by LAD showing high cytoplasmic expression of galectin-1 by cardiac myocytes (thin arrow) surrounding areas of low or absence of expression (star shape), streptavidin- biotin immunoperoxidase method. H, I& J, Representative section from left ventricle showing a well demarcated area of high cytoplasmic expression of galectin-1 by cardiac myocytes (thin arrow), Alexa Fluor 488 immunofluorescent technique.

**Figure 9:**
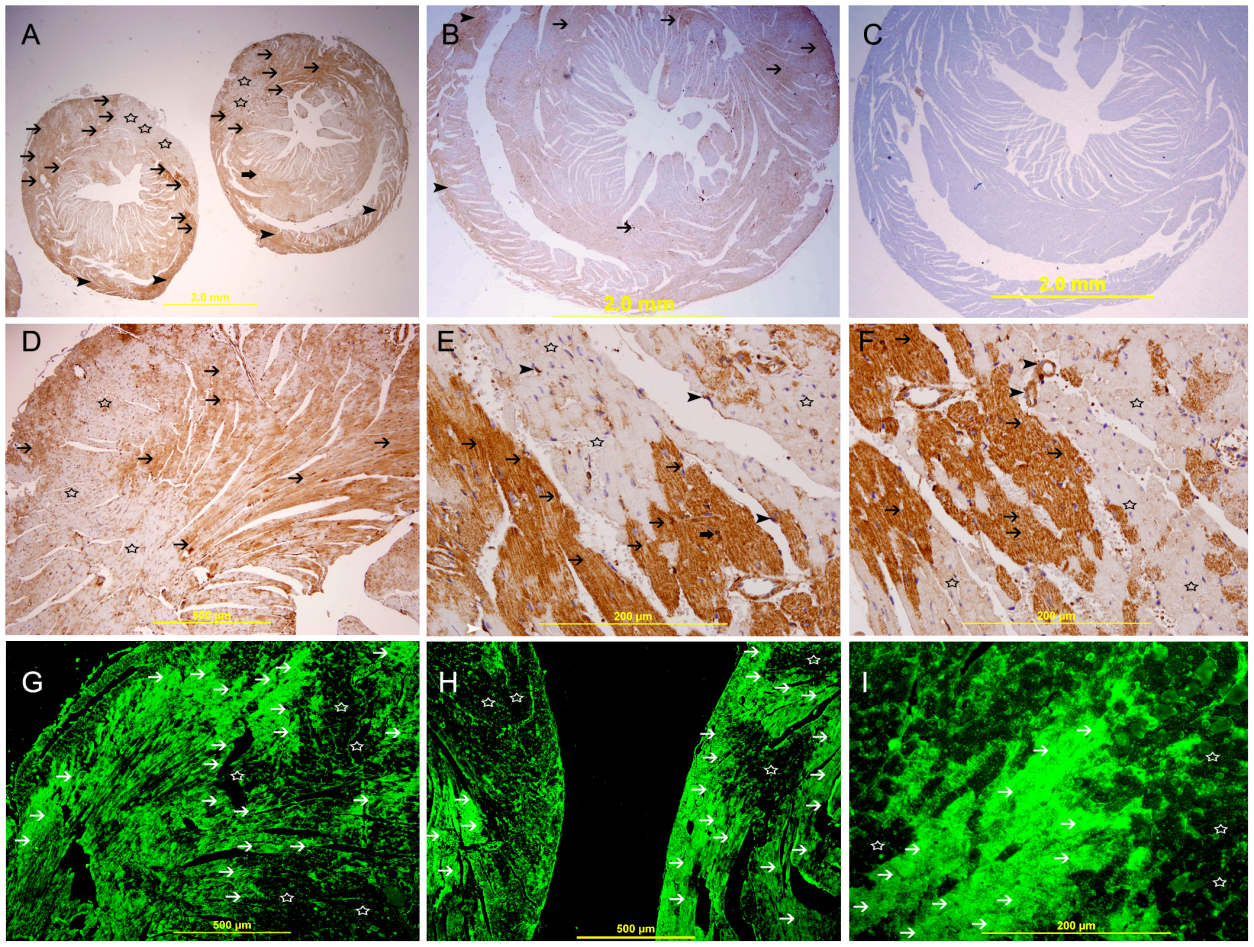
Galectin-1 expression 4 hours following ligation of LAD. A.A low power view of the heart showing a high expression of galectin-1 in the anterior wall of left ventricle in the area supplied by LAD (thin arrow) and the interventricular septum (thick arrow). There is also increase in the expression of galectin-1 in the right ventricle (arrow head), streptavidin- biotin immunoperoxidase method. B. Sham operated heart showing low expression of galectin-1 in the left ventricle (thin arrow) and right ventricle (arrow head), streptavidin- biotin immunoperoxidase method. C. Negative control section of the heart showing absence of staining of galectin-1. D. Representative section of the left ventricle from area supplied by LAD showing high cytoplasmic expression of galectin-1 by cardiac myocytes (thin arrow) surrounding areas of low or no expression (star shape), streptavidin- biotin immunoperoxidase method. E&F, Representative section of the left ventricle from area supplied by LAD showing a well demarcated area of high cytoplasmic expression of galectin-1 by cardiac myocytes (thin arrow), streptavidin- biotin immunoperoxidase method. G, H&I. Representative section from left ventricle showing a well demarcated area of high cytoplasmic expression of galectin-1 by cardiac myocytes (thin arrow) surrounding areas of low or no expression (star shape), Alexa Fluor 488 immunofluorescent technique.

**Figure 10:**
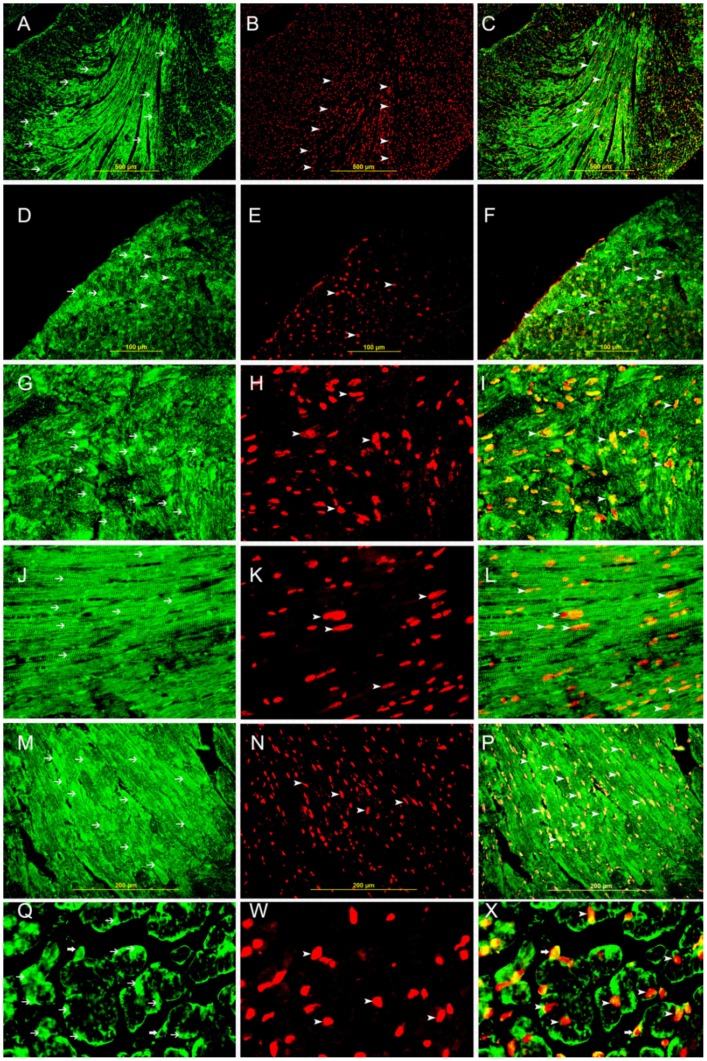
Co-localization of Galectin-1 and HIF-1 α. A,D,G,J,M, Q show representative sections of the left ventricle from areas supplied by LAD in 20 minutes(Low power view), 20 minutes(high power view), 30 minutes, 60 minutes, 4 hours and 30 minutes (High power view) following MI respectively, showing high cytoplasmic expression of galectin-1 by cardiac myocytes (thin arrow), Alexa Fluor 488 immunofluorescent technique. B,E,H,K,N,W show representative sections of the left ventricle from areas supplied by LAD in 20 minutes(Low power view), 20 minutes(high power view), 30 minutes, 60 minutes, 4 hours and 30 minutes (High power view) following MI respectively showing high nuclear expression of HIF-1α by cardiac myocytes (thin arrow), Rhodamine immunofluorescent technique. C,F,I,L,P,X shows Co-localization of galectin-1 and HIF-1 α in the same sections at the respective time points. (arrow head), Alexa Fluor-Rhodamine immunofluorescent technique.

### Galectin-1 Levels in Plasma

Plasma GAL-1 concentration in naïve group is 8.71±0.83 ng/ml. Plasma GAL-1 concentration is significantly raised at 4 hours (15.05±1.09 vs 9.91±1.35 ng/ml, ***P*** = 0.012*) and 24 hours (22.59±0.42 vs 17.86±0.93 ng/ml, ***P =*** **0.001** *) post MI groups compared to corresponding sham operated control groups (**Fig. 2 D**). Plasma GAL-1 values at 30 minutes and 60 minutes post MI groups are not significantly different from their corresponding sham-operated control groups (15.49±1.29 vs 14.06±0.78 ng/ml and 14.27±0.72 vs 13.33±1.49 ng/ml).

### Co-localization of Galectin-1 and HIF-1 α

**Figure 11:**
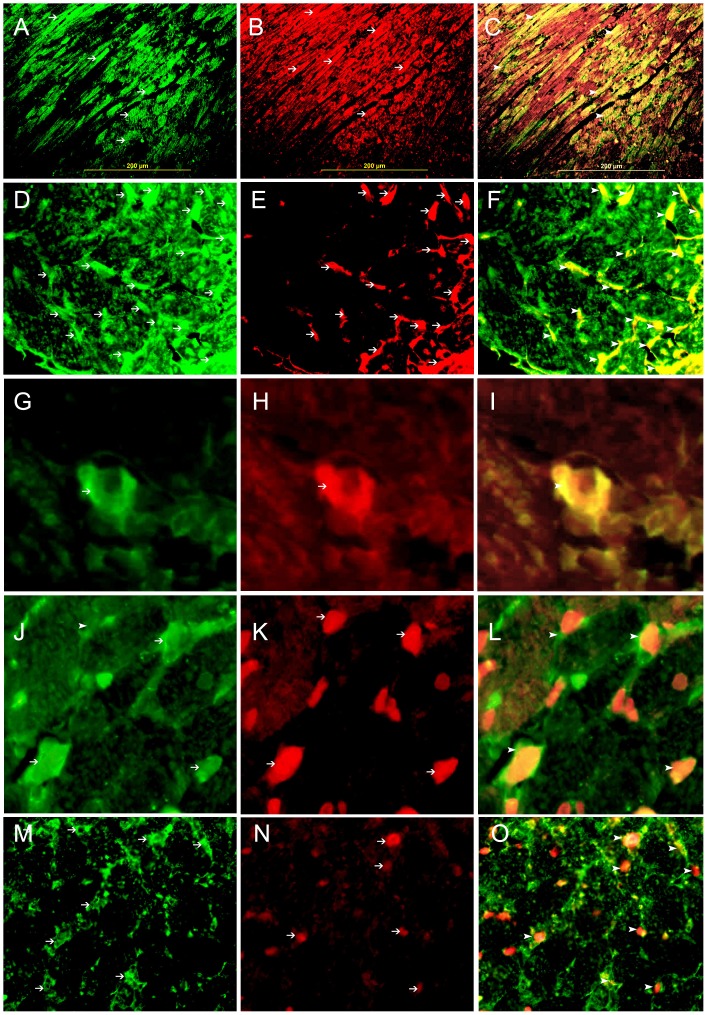
Co-localization of Galectin 1, HIF-1 α, CD31, desmin and CD68. A,D,G, Representative section of the left ventricle from areas supplied by LAD showing high cytoplasmic expression of galectin-1 by cardiac myocytes, endothelial cells and histiocytes respectively (thin arrows). J, showing high cytoplasmic expression of CD68 in tissue histiocytes (thin arrows) and M showing high cytoplasmic expression of CD31 in endothelial cells (thin arrows). Alexa Fluor 488 immunofluorescent technique. B. showing high cytoplasmic expression of desmin by cardiac myocytes (thin arrow), E. showing high cytoplasmic expression of CD31 by endothelial cells (thin arrow), H. showing high cytoplasmic expression of CD68 by histiocytes (thin arrow), K. showing high nuclear expression of HIF-1 α by histiocytes (thin arrow), N. showing high nuclear expression of HIF 1 α by endothelial cells (thin arrow), Rhodamine, immunofluorescent technique. C. Co-localization of galectin-1 and desmin in cardiac myocytes. F. Co-localization of galectin-1 and CD31 in endothelial cells (arrow head), I. Co-localization of galectin-1 and CD68 in histiocytes (arrow head), L. Co-localization of CD68 and HIF-1 α in tissue histiocytes (arrow head), O. Co-localization of CD31 and HIF-1 α in endothelial cells (arrow head). Alexa Fluor 488-Rhodamine immunofluorescent technique.

Co-localization of GAL-1 and HIF-1 **α** in cardiomyocytes and endothelial cells is shown in **Fig. 10**. All cardiac myocytes that show nuclear expression of HIF-1 α also express GAL-1 in the cytoplasm and some nuclei. All endothelial cells that show nuclear expression of HIF-1 α also express GAL-1 in the cytoplasm and some nuclei. GAL-1 expression in cardiomyocytes is confirmed by co-localizing it with Desmin **Fig. 11 C**. GAL-1 and HIF-1 **α** expression in endothelial cells is shown with co-localization with CD31 (**Fig. 11F&O** **respectively**). Few tissue histiocytes, which express CD68, also show co-localization with GAL-1 and HIF-1 **α** (**Fig. 11 I& L** **respectively**). In general, few tissue histiocytes are noticed during the first 24 hours following MI.

### Morphometric Analysis

The frequency of cardiomyocytes expressing GAL-1 in 20 and 30 minutes post MI group is higher than other MI groups and show statistically significance when compared with 24-hour post MI group (Chi squared = 6.779 with 1 degree of freedom. P = 0.009* and Chi squared = 9.968 with 1 degree of freedom. P = 0.001*, respectively). The frequency of endothelial cells expressing GAL-1 in 20 is higher than other MI groups and shows statistical significance when compared with 60 minutes, 4-hour and 24-hour post MI groups (Chi squared = 4.282 with 1 degree of freedom. P = 0.03*, Chi squared = 6.627 with 1 degree of freedom. P = 0.01*, Chi squared = 9.29 with 1 degree of freedom. P = 0.002*, respectively). The frequency of endothelial cells expressing GAL-1 in 30 minutes post MI group is significantly higher than 24-hour post MI groups (Chi squared = 3.931 with 1 degree of freedom. P = 0.047*). The frequency of cardiomyocytes expressing HIF-1 α in 20 minutes post MI group is significantly higher than 30 minutes, 60 minutes, 4-hour and 24-hour post MI groups (Chi squared = 9.158 with 1 degree of freedom. P = 0.002*, Chi squared = 34.1 with 1 degree of freedom. P = 0.0001*, Chi squared = 57.001 with 1 degree of freedom. P = 0.0001*, Chi squared = 84.5 with 1 degree of freedom. P = 0.0001*, respectively). The frequency of endothelial cells expressing HIF-1 α in 20 minutes post MI group is significantly higher than 30 minutes, 60 minutes, 4-hour and 24-hour post MI groups (Chi squared = 12.3 with 1 degree of freedom. P = 0.0004*, Chi squared = 36.5 with 1 degree of freedom. P = 0.0001, Chi squared = 49.1 with 1 degree of freedom. P = 0.0001, Chi squared = 54.3 with 1 degree of freedom. P = 0.0001*, respectively). The frequency of endothelial cells expressing GAL-1 is significantly higher than cardiomyocytes at 20 minutes, 30 minutes, 60 minutes, 4-hour and 24-hour post MI groups (Chi squared = 42.4 with 1 degree of freedom. P = 0.0001*, Chi squared = 25.9 with 1 degree of freedom. P = 0.0001*, Chi squared = 31.1 with 1 degree of freedom. P = 0.0001*, Chi squared = 34.3 with 1 degree of freedom. P = 0.0001*, Chi squared = 38.8 with 1 degree of freedom. P = 0.0001*, respectively). The frequency of endothelial cells expressing HIF-1 α is significantly higher than cardiomyocytes at 4-hour and 24-hour post MI groups (Chi squared = 6.84 with 1 degree of freedom. P = 0.008* and Chi squared = 17.04 with 1 degree of freedom. P = 0.0001*, respectively). The frequency of neutrophil polymorphs expressing GAL-1 is significantly higher than cardiomyocytes and endothelial cells at 24-hour post MI groups (Chi squared = 69.8 with 1 degree of freedom. P = 0.0001* and Chi squared = 5.72 with 1 degree of freedom. P = 0.016*, respectively). The frequency of neutrophil polymorphs expressing HIF-1 α is significantly higher than cardiomyocytes and endothelial cells at 24-hour post MI groups (Chi squared = 81.9 with 1 degree of freedom. P = 0.0001* and Chi squared = 27.9 with 1 degree of freedom. P = 0.0001*, respectively). A decrease in the number of cardiomyocytes and endothelial cells that express GAL-1 and HIF-1 α is associated with the increase in post MI time. Neutrophil polymorphs were counted at 24-hour following MI as they are not seen before 4 hour post MI time (**Table 4**).

**Table 4:** Morphometric analysis of expression of GAL-1 and HIF-1α in cardiomyocytes, endothelial cells and neutrophil polymorphs at different time points following ligation of LAD.

POST MI	GALECTIN -1			HIF-1α		
TIME POINTS	Cardiomyocytes %	Endothelial cells %	Neutrophils %	Cardiomyocytes %	Endothelial cells %	Neutrophils %
20 MINUTES	49	92	0	83	94	0
30 MINUTES	53	87	0	63	75	0
60 MINUTES	41	81	0	42	56	0
4 HOURS	36	78	0	29	48	0
24 HOURS	30	75	89	17	45	82

### Proliferation and Apoptosis in Early Post Myocardial Infarction

**Figure 12:**
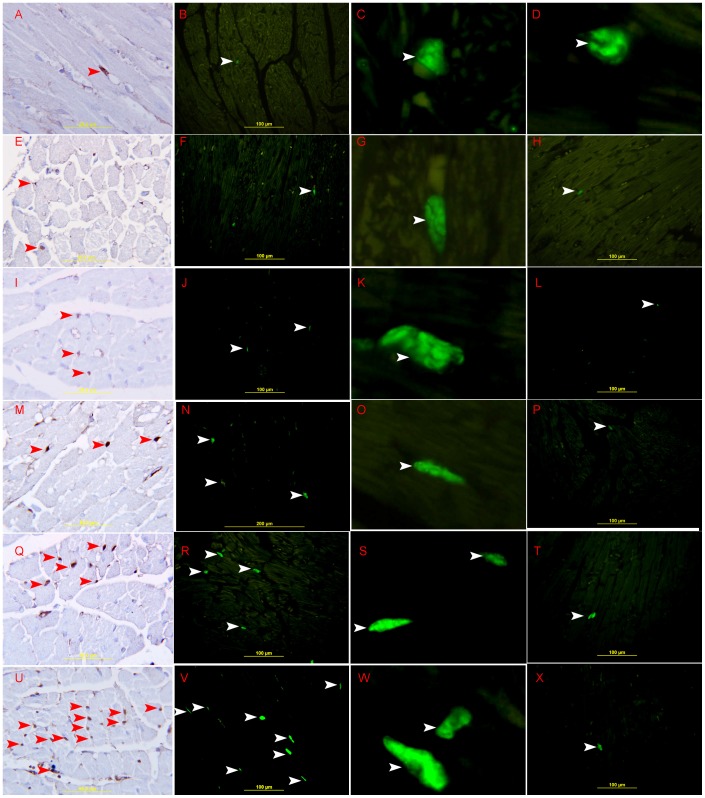
Ki-67 proliferative activity in the left ventricle. A, B, C, D, Low Ki-67 proliferative activity in normal left ventricle showing nuclear staining of Ki-67 in one interstitial cell (arrow head). E, F, G, 20 minutes MI; I,J, K, 30 minutes MI; M,N,O 60 minutes MI; Q,R,S, 4-hour MI, Low Ki-67 proliferative activity: showing nuclear staining of Ki-67 in few endothelial cells (arrow head) in left ventricle. U, V, W, 24-hour MI, high Ki-67 proliferative activity showing nuclear staining of Ki-67 in a large number of endothelial cells (arrow head) in the infarction area of left ventricle. H, L, P, T, X, showing faint nuclear staining of Ki-67 in one endothelial cell in 20 minutes, 30 minutes, 60 minutes, 4-hours and 24 hour sham-operated left ventricle. A, E, I, M, Q, U are stained by Streptavidin-Biotin immunoperoxidase method. The others are stained by Alexa Fluor 488 immunofluorescent technique.

Our staining with Ki-67 showed that there is a low proliferative activity in 20 minutes, 30 minutes, 60 minutes and 4-hour post MI sections. **(** **Figure 12** **)**, but in 24 hours post MI sections we are able to see clearly an increase in the expression of Ki-67 in the endothelial cells in the area of infarction **(** **Figure 12 U&V** **)** while the relevant sham operated sections show very low expression of Ki-67 **(** **Figure 12 X** **)**.

**Figure 13:**
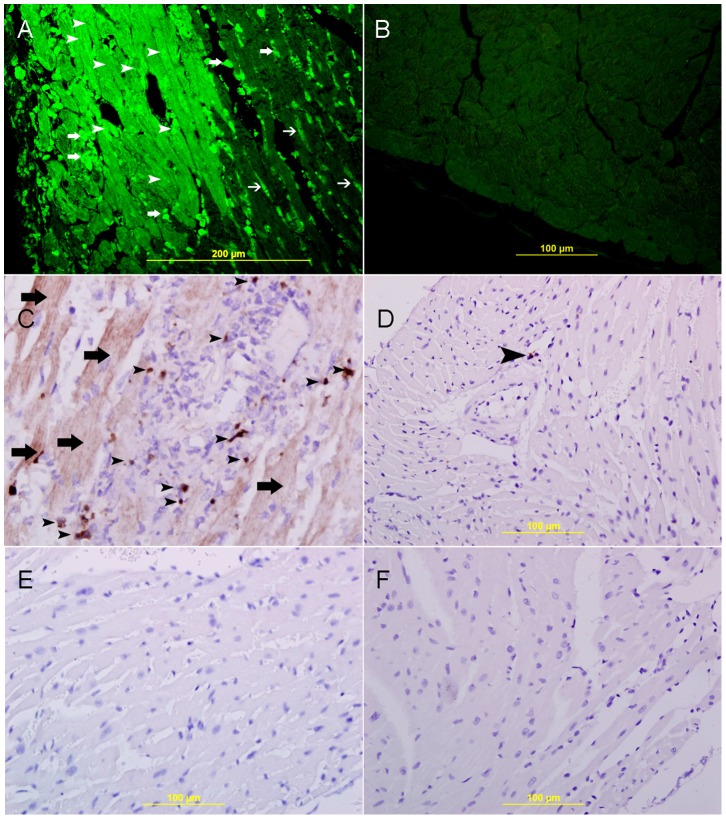
Apoptotic activity in the left ventricle. A. showing high expression of caspase- 3 in_cardiac myocytes (arrow head) surrounding the necrotic area, endothelial cells (thin arrow), and neutrophil polymorphs (thick arrow) in 24-hour post MI, Alexa Fluor 488 immunofluorescent technique. B. showing no expression of caspase 3 in 24-hour sham-operated left ventricle. C, showing high expression of cleaved caspase- 3 in cardiac myocytes (thick arrows). Many apoptotic bodies (arrow head) are seen in the infarcted area of left ventricle, streptavidin- biotin immunoperoxidase method. D, showing very low apoptotic activity, only one cell stained with anti-cleaved caspase 3 (arrow head) in the area of infraction in the left ventricle of 4-hour post MI, streptavidin- biotin immunoperoxidase method. E, F, Showing no staining with anti-cleaved caspase 3 in the left ventricle of 30 minutes and 60 minutes post MI, streptavidin- biotin immunoperoxidase method.

We also found that there is very low expression of caspase-3 and cleaved caspase-3 at 30 minutes, 60 min and 4 hours time points **(** **Figure 13 D, E, F** **)**. At 24- hour post MI group however, we do find increase in the expression of caspase-3 and cleaved caspase-3 activity **(** **Figure 13 A, C** **)**, compared to the sham group **(** **Figure 13 B** **)**. The expression of caspase-3 and cleaved caspase 3 was seen in cardiac myocytes and endothelial cells in the area of infarction. Many apoptotic bodies, expressing cleaved caspase-3, are seen in the area of infarction **(** **Figure 13 C** **)**.

**Figure 14:**
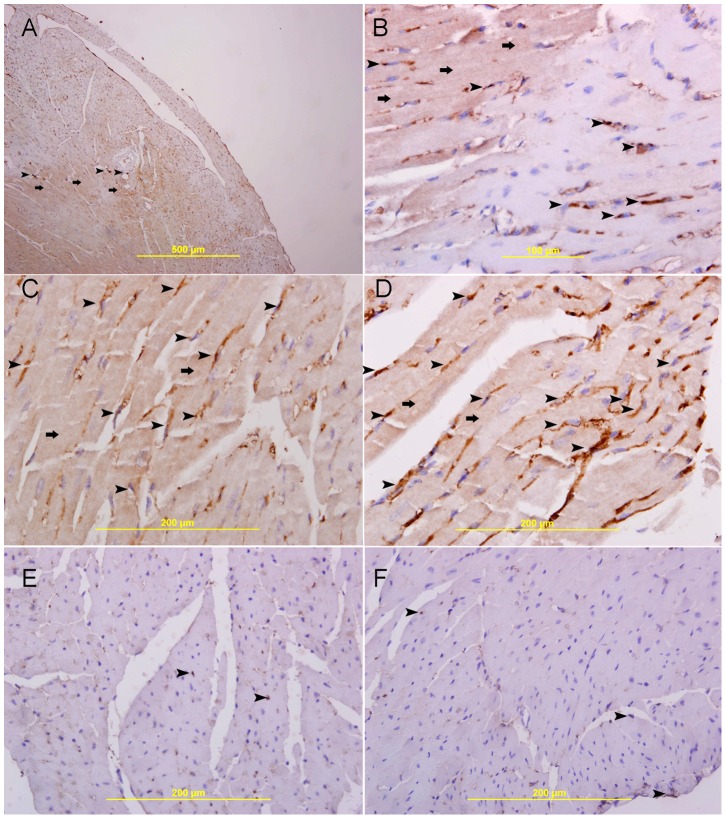
Bcl2 activity in left ventricle. A, B, C, D, showing high cytoplasmic expression of bcl2 by cardiac myocytes (thick arrow), and endothelial cells (arrow head) at 30 minutes post MI in an area supplied by LAD in the left ventricle, streptavidin- biotin immunoperoxidase method. E, F, showing very low expression of bcl2 in few endothelial cells (arrow head) in the left ventricle of naïve and 30-minutes sham operated heart. Streptavidin- biotin immunoperoxidase method.

Bcl-2 expression is increased in the 30 minutes post infarction time in the LV in the area of infarction of the left ventricle (**Figure 14 A–D**). Its expression is seen in the cardiomyocytes and endothelial cells; however, the expression in the endothelial cells is considerably greater than the cardiomyocytes. Sham group showed faint staining of bcl-2 and only in the endothelial cells.

### Bioinformatic Analysis Results

**Figure 15:**
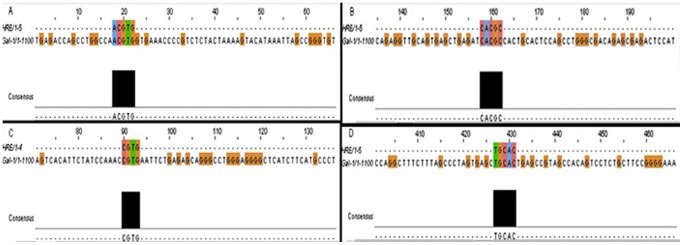
Potential HREs in the promoter region of galectin-1 gene. A, Human GAL-1 promoter with consensus sequence ACGTG (-1075). B, Human GAL-1 promoter with consensus sequence CACGC (−519, −533, −935). C, Mouse GAL-1 promoter with consensus sequence NCGTG (−978). D, Mouse GAL-1 promoter with consensus sequence TGCAC (−640).

The output from the clustalw2 program is shown in **Fig. 15**. We found at least 4 potential HREs in the promoter region of Human GAL-1 gene (**Fig. 15 A & B**) and potential HRE’s in the promoter region of mouse GAL-1 gene (**Fig. 15 C&D**).

## Discussion

Galectins are highly conserved, from fungi to mammals with their existence dating back to more than 800 million years. This suggests that these endogenous proteins must be serving an important purpose [31]–[33]. Although GAL-1 knockout mice fail to show any abnormalities [11], but knocking down GAL-1-like protein in the zebrafish shows defects in muscle development and disorganized muscle fibers [34]. There is also evidence that GAL-1 has a significant role in the regeneration of muscles [35]–[37].

GAL-1 is endogenous to the heart. The level of expression in cardiomyocytes is among the highest compared to other organs of the body [38]. In accordance with a previous report [38], our immunohistochemistry and immunoflorescence staining of heart sections show that it is expressed in cardiac muscles as diffuse cytoplasmic staining, as an organized Z bands staining, as a membranous staining and as nuclear staining.

Acute MI in our mice model shows increase in the level of GAL-1 in the LV at a very early stage in the course of event. We show a significant increase in LV tissue GAL-1 levels at 20 minutes and 30 minutes following MI as compared to related sham operated control groups. The values at 60 minutes and 4 hours are also higher than their corresponding sham operated control groups but do not reach statistical significance (Figure 2 C). These results show that there is a transient rise in the GAL-1 level in the LV within one hour of permanent ligation of LAD. This is supported by one-way ANOVA and Tukey post-hoc test analysis of MI groups which shows GAL-1 values at 20 and 30 minutes MI group are significantly higher than other MI groups.

The immunohistochemical and immunofluorescent staining patterns for GAL-1 in cardiac muscles are also very characteristic and supportive of this finding. The increased expression of GAL-1 in the LV is very well demarcated in the MI group in all tested time points. We also noticed that as the time of ischemia increases from 20 min to 4 hours and then 24 hours we are able to recognize areas of low or no expression of GAL-1 being surrounded by areas of high expression of GAL-1. The well demarcated areas of no or low expression of GAL-1 is increased as the time of ischemia increases which mean that as time proceed dying cells will stop expression of GAL-1 in the area supplied by LAD, while survived cells in the ischemic zone, which are seen at the periphery of infarction zone, are showing high expression of GAL-1; which might explain the absence of statistical significance at 60 minutes, 4-hour and 24-hour post MI time points when compared to corresponding sham operated control groups. This is supported by our morphometric analysis which shows a significant decrease in the frequency of cardiomyocytes and endothelial cells that express GAL-1 with the increase of post MI time.

This phenomenon is clearly seen in 24 hours post MI sections (**Fig. 4 E, F, G, H, &I**) where GAL-1 is observed to be high in the surviving cardiomyocytes and invading neutrophil polymorphs in the immunohistochemical and immunofluorescent- stained sections, while dead cells in the infarction zone do not show any expression which explains the absence of statistical significance in our ELISA results at this time point.

GAL-1 has been reported to be up regulated in tumors under hypoxic conditions [20]. It also plays a role in chronic hypoxia in the lung [22], and has shown to be important in cerebral, lung and renal ischemic events [21], [22], [39], [40]. One recent study reported raised GAL-1 levels in HL-1 cardiomyocytes exposed to hypoxic environment [41]. The same study also showed raised GAL-1 concentration in the hearts of mice after 7 days of undergoing experimental acute myocardial infarction emphasizing on its role in the post infarction remodeling of the heart [41]. They have suggested a protective role for GAL-1 through its negative regulation of the heart’s inflammatory response. We report for the first time that GAL-1 is elevated in the LV very early following acute myocardial infarction. The first 4 hours post MI is the time before inflammatory cells particularly neutrophil polymorphs flood the infarcted area of the heart [42]. So the changes observed till 4- hour post MI are not due to the effect of invading inflammatory cells but predominantly due to hypoxia/ischemia related factors, while the changes at 24 hours following MI time can be a combination of inflammatory and hypoxia/ischemia related players.

HIF-1 α levels in the LV also shows a significant increase in 20 minutes post MI group compared to sham operated control group **(** **Fig. 2 A&B** **)**. In addition, HIF-1 α value in the MI groups at 30 minutes, 1 hour and 4 hours are higher than corresponding sham operated control groups but shows no statistical significance. This is supported by one-way ANOVA and Tukey post-hoc test analysis of MI groups which shows HIF-1 α value at 20 minutes MI group is significantly higher than other MI groups. Moreover, HIF-1 α values in all MI groups is higher than the baseline non-operated naïve control groups. We show for the first time a transient peak in LV HIF-1 α level at 20 minutes following MI, which goes down afterwards.

The immunohistochemical and immunofluorescent staining results are very characteristic and supportive of this pattern **(** **Fig. 3** **)**.

We know that HIF-1 α is a heterodimeric DNA-binding complex composed of constitutively expressed HIF-1β or aryl hydrocarbon nuclear translocator and the oxygen sensitive hypoxia-inducible HIF-1α [43]. Under normoxic conditions HIF-1α subunit has a very short half-life [44] and is continuously undergoing proteasomal degradation. During hypoxia, HIF-1α protein escapes proteasomal degradation, translocates to the nucleus and dimerizes with HIF-1β. This complex then binds to the HRE in promoter or enhancer sequences of target genes [45] and results in their transcription.

We believe that in our experiments, initial increase in HIF-1 α levels in the LV is due to stabilization of HIF-1 α as a result of low intracellular level of oxygen secondary to complete ligation of LAD artery. We also observe a decrease in LV level of HIF-1 α as the time of ischemia increased. We think that as the time of ischemia increases the cardiomyocytes become necrotic and HIF-1 α level go down due to protein degradation. As mentioned in the methods section, we took only the LV protein extraction, so the mass of heart tissue is more or less similar between samples. If the cells in the middle of the infarct are necrotic and HIF- 1 α is degraded as the time of ischemia increases then it is understandable that the levels of HIF-1 α in the 1 hour, 4 hour and 24 hour post MI groups are not significantly higher than corresponding sham operated groups.

HIF-1 α is being expressed by the nuclei of cardiomyocytes and endothelial cells follows the same pattern seen in immunohistochemical and immunofluorescent-stained sections and we are able to see that as the time of ischemia increases the number of cells with high expression of HIF-1 α is decreased. This is supported by our morphometric analysis which shows a significant decrease in the frequency of cardiomyocytes and endothelial cells that express HIF-1 α with the increase of post MI time.

Another observation pertinent to HIF-1 α staining was that while the expression of HIF-1 α decreases in cardiomyocytes as the time of ischemia increases, its expression in endothelial cells essentially remains the same. A possible explanation for this can be related to the fact that endothelial cells are proliferating cells that participate in healing process of the infarcted zone and in the formation of collaterals while survived cardiomyocytes do not proliferate. We think that when continuous ischemia damages the cardiomyocytes its expression is lost but the surrounding endothelial cells keep on proliferating and expressing HIF-1 α.

HIF-1 α is a protective factor that mediates the survival of injured cardiomyocytes in the setting of ischemic injury by transcribing a variety of cardioprotective genes including erythropoietin, vascular endothelial growth factor, inducible nitric oxide synthase, hemeoxygenase-1 and cardiotropin [46].

In **Fig. 10** and **11**, we show co-localization of GAL-1 and HIF-1 α in cardiac myocytes and endothelial cells in LV sections from areas supplied by LAD at different time points in the first 4 hours following MI. Cardiomyocytes and endothelial cells that show nuclear expression of HIF-1 α also show cytoplasmic and nuclear expression of GAL-1, while cells that do not express HIF-1 α they also show no expression of GAL-1, which might indicate a possible correlation in the expression of both proteins. In **Fig. 4 K, L, M**, we are able to show co-expression of GAL-1 and HIF-1 α in surviving cardiomyocytes and endothelial cells at the periphery of infarction zone while dead cardiomyocytes in the centre of infarction do not show any expression, which might also support a possible correlation between both proteins. In the same figure, we are also able to show co-expression of GAL-1 and HIF-1 α by neutrophil polymorphs that infiltrate the myocardium following MI to digest dead cells and facilitate their removal at a later time by macrophages. Those neutrophil polymorphs are moving in between dead cells in the ischemic zone, so they are expected to be under hypoxic condition; therefore they are expressing HIF-1 α; as hypoxia can stabilize HIF-1 α and prevents its proteasomal degradation. At the same time, we notice those invading neutrophil polymorphs are also expressing GAL-1, which might also support a possible correlation.

In **Fig. 2 E**, we show the pattern of GAL-1 and HIF-1 α in the heart from 20 minutes till 24 hours post MI time points. It shows both proteins follow the same pattern in the first 24 hours following MI and their co-localization seen by immunofluorescent staining further supports our idea that GAL-1 is a possible transcriptional target of HIF-1 α in the heart at least in the early period after MI.

We also found potential HRE’s in the promoter region of GAL-1 through bioinformatics analysis **(** **Fig. 15** **)**. This is supported by a previous study [23], which also showed that there are seven potential HREs within 2.2 kb region upstream the transcriptional start site of GAL-1. Experimental evidence also exists of GAL-1 being a transcriptional target of HIF-1 α [23].

We suggest that in our experiment, GAL-1 gene transcription by HIF-1 α can lead to increased expression of GAL-1 in cardiomyocytes, which might also prove to be cardioprotective due to its anti-inflammatory properties [47], [48]. As the time of ischemia increases, cells in the centre of the infarct loose HIF-1 α and consequently GAL-1 while the surrounding surviving cells express high levels of HIF-1 alpha and subsequent GAL-1 to limit damage and prevent further injury.

To further look into the biological role underlying the early expression of GAL-1 and HIF-1 α in cardiomyocytes, we looked at the pro-apoptotic and anti-apoptotic proteins at these early time points. Pro-apoptotic caspase-3 and cleaved caspase-3 were found to have a very low expression at early time points post MI, while in 24-hour post MI group the expression is increased in the area of infarction compared to the sham operated group **(** **Fig. 13** **)**. Moreover, the anti-apoptotic Bcl-2 expression is high at early time points, especially in 30 minutes post MI group, in the area of infarction **(** **Fig. 14** **)**. As we can see that in the early post MI time points there is predominantly antiapoptotic activity in the left ventricle which correlates with the high tissue GAL-1 and HIF-1 α levels at that time. While in 24-hour post MI time point we have high apoptotic activity which correlates with low tissue levels of GAL-1 and HIF-1 α at that time. This further supports our concept that GAL-1 and HIF-1 α are part of the prosurvival mode of action of the cell after ischemic insult at least in the early myocardial infarction time.

We also found that in the early post MI time there is low proliferative activity in the LV (**Fig. 12**), so we are unable to comment on any proliferative role of GAL-1 and HIF-1 α at these time points. While in 24-hour post MI group there is an increase in the proliferative activity of endothelial cells around the area of infarction (**Fig. 12 U&V**) as part of the attempts to increase vascularity in the ischemic area to overcome low perfusion as well as participating in the healing process [49].

Another significant finding of our study is that GAL-1 plasma level is significantly high around 4 hours and 24 hours post MI compared to sham operated control mice **(** **Fig. 2 D** **)**.

There can be two possible explanations for this phenomenon. We know that GAL-1 is present in the heart tissue in significant amount, acute myocardial infarction and subsequent cell membrane damage and necrosis promotes its leakage outside the cells into the blood. This phenomenon is similar to the raised Troponin-I protein [50] that escapes the injured cardiomyocytes and can be detected in the blood. The other explanation for raised plasma levels is that GAL-1 in heart tissue is increased in early ischemic period due to increased transcriptional pressure from HIF-1 α and is secreted from the heart through the non-classical pathway. We think that plasma GAL-1 levels in the first hour post MI signify increase in transcription of the protein and leakage from the cells, whereas, at 4 hours and 24 hours post MI, the high GAL-1 plasma levels is mainly due to leakage of this protein from cardiomyocytes.

We have observed in our study that the levels of GAL- 1 and HIF-1 α in sham operated animals were higher than the non- operated naïve animals. This increase in sham operated animals can be due to factors related to surgical stress. There have been reports in scientific literature that there is stress-induced increase of GAL- 1 in serum which is regulated by the sympathetic nervous system [51]. Another study [52] demonstrated that HIF-1 α protein in the myocardium can be induced by mechanical stress to the heart. As there is some degree of mechanical/surgical stress applied to the sham operated mice we can suggest that GAL-1 and HIF-1 α levels seen in sham group may be the result of these factors.

For these reasons we make sham operated groups as our controls for all time points, and all our statistics were done in comparison between MI groups and sham-operated groups to take out the effect of mechanical/surgical stress from the real ischemic effect due to ligation of LAD. In all early MI groups the values of GAL-1 and HIF-1 are higher than corresponding sham groups and show statistical significance at certain time points. So the significant rise of GAL-1 and HIF-1 α levels at certain time points in MI groups when compared to sham-operated groups is purely due to ischemia and not surgical stress.

## Conclusions

We show for the first time that GAL-1 level in the LV is increased in early ischemic period which can possibly be a part of the prosurvival gene expression profile transcribed by HIF-1 α. We also report for the first time that HIF-1 α is significantly increased at 20 minutes following myocardial infarction. In addition, we report for the first time that in mouse model of myocardial infarction plasma GAL-1 level is significantly raised as early as 4 hours of the event.

The study is significant because it can help in understanding the very early changes that occur in the myocardium after acute infarction and help devise ways to diagnose early and save the viable tissue before permanent damage sets in. Raised plasma GAL-1 levels in this early phase of infarction in the mice is significant in terms of its potential use as a biomarker for MI, but further studies are still needed to elaborate on its role.
